# The Biological Functions and Clinical Applications of Integrins in Cancers

**DOI:** 10.3389/fphar.2020.579068

**Published:** 2020-09-11

**Authors:** Chao-yue Su, Jing-quan Li, Ling-ling Zhang, Hui Wang, Feng-hua Wang, Yi-wen Tao, Yu-qing Wang, Qiao-ru Guo, Jia-jun Li, Yun Liu, Yan-yan Yan, Jian-ye Zhang

**Affiliations:** ^1^ The Fifth Affiliated Hospital, Key Laboratory of Molecular Target and Clinical Pharmacology and the State Key Laboratory of Respiratory Disease, School of Pharmaceutical Sciences, Guangzhou Medical University, Guangzhou, China; ^2^ The First Affiliated Hospital, Hainan Medical University, Haikou, China; ^3^ Guangzhou Institute of Pediatrics/Guangzhou Women and Children’s Medical Center, Guangzhou Medical University, Guangzhou, China; ^4^ Institute of Immunology and School of Medicine, Shanxi Datong University, Datong, China

**Keywords:** integrins, cancer metastasis, drug resistance, stemness, extracellular matrix, therapeutic targeting

## Abstract

Integrins are the adhesion molecules and receptors of extracellular matrix (ECM). They mediate the interactions between cells-cells and cells-ECM. The crosstalk between cancer cells and their microenvironment triggers a variety of critical signaling cues and promotes the malignant phenotype of cancer. As a type of transmembrane protein, integrin-mediated cell adhesion is essential in regulating various biological functions of cancer cells. Recent evidence has shown that integrins present on tumor cells or tumor-associated stromal cells are involved in ECM remodeling, and as mechanotransducers sensing changes in the biophysical properties of the ECM, which contribute to cancer metastasis, stemness and drug resistance. In this review, we outline the mechanism of integrin-mediated effects on biological changes of cancers and highlight the current status of clinical treatments by targeting integrins.

## Introduction

The transformation process from normal cells to malignant cancer cells involves a series of complex pathological mechanisms, including the abnormal activation/deactivation of various cancer-related signaling molecules and signaling pathways (Cooper and Giancotti, 2019). Incipient cancer cells acquire multiple biological functions during their evolution that enable them to become tumorigenic and ultimately malignant (Hanahan and Weinberg, 2011). Integrins are widely present on the surface of cells and mediate the adhesion between cells -to -cells and cells to ECM (Hamidi and Ivaska, 2018). Accumulating evidence showed that integrins and integrin-dependent biological process play vital roles in mediating cancer stem-like property, cancer metastasis and drug resistance (Seguin et al., 2015; Hamidi et al., 2016; Cooper and Giancotti, 2019). Interaction between integrins and ECM enhances cell adhesion and activates cancer cell pro-survival and anti-apoptotic programs, resulting in the development of drug resistance (Leask, 2019). In addition, integrins are involved in the regulation of survival signaling of cancer stem cells (CSCs), which is another reason for developing cancer drug resistance (Seguin et al., 2015). A number of studies in recent years have reported that integrins on exosomes make a significant contribution in mediating cancer organotropic metastasis and preparing pre-metastatic niche (Hoshino et al., 2015; Paolillo and Schinelli, 2017; Shimaoka et al., 2019). Hoshino et al. (2015) first demonstrated that tumor exosomal integrins mediated organotropic metastasis. Given the multiple biological functions mediated by integrins in cancers, integrins have been regarded as a promising target for cancer treatment. Although there are few successful clinical trials, many preclinical studies have shown encouraging results (Hamidi and Ivaska, 2018). Additionally, integrins, such as integrin αvβ3, integrin α6 and integrin α7 might have potential as cancer diagnostic and prognostic biomarkers (Seguin et al., 2015; Haas et al., 2017). In this review, we summarized current studies on the roles of integrins in cancer progression and its clinical value.

## Integrins: An Overview

Integrins consist of 18 α and 8 β subunits, that pair to form at least 24 different functional heterodimeric receptors (Humphries et al., 2006). Integrin heterodimers are transported from the endoplasmic reticulum to Golgi apparatus, where they are further post-translationally modified and transferred to the cell surface in an inactive state (De Franceschi et al., 2015). The integrin α and β subunits are both glycosylated, and their amino acid terminals are bonded to each other by a non-covalent bond, thereby forming αβ integrin heterodimers (Seguin et al., 2015). Some integrin subunits only appear in a single heterodimer, 12 integrins contain β1-subunits and five contain αv-subunits (Kechagia et al., 2019). As a receptor on the cell membrane, integrins mainly interact with ECM components to mediate cell adhesion (Dustin, 2019). According to different types of ECM components, integrins can be classified into two main categories: receptors that recognize Arg-Gly-Asp (RGD) peptide motifs and receptors that independent on RGD binding region (collagen receptors, laminin receptors and leukocyte-specific integrins) (Hamidi and Ivaska, 2018). On one hand, different types of integrins can recognize and bind the same ligand (Kechagia et al., 2019). For example, all five αv integrins (αvβ1, αvβ3, αvβ5, αvβ6, and αvβ8) and two β1 integrins (α5β1 and α8β1) and αIIbβ3 are RGD-binding integrins (Humphries et al., 2006). Integrins α1β1, α2β1, α10β1, and α11β1 binding to laminins and collagens (Humphries et al., 2006). The common feature of these integrins is that they contain an α-subunit of the αA-domain, which specifically bind to β1-subunit. Additionally, three β1 integrins (α3β1, α6β1, and α7β1) and α6β4 are highly selective laminin receptors (Marsico et al., 2018). Interestingly, the α-subunits of these integrins do not contain αA-domain (Marsico et al., 2018). Moreover, α4β1, α4β7, α9β1, and αEβ7 recognize similar sequences in their ligands. On the other hand, the same integrins can bind to multiple ligands (Kechagia et al., 2019). For instance, αvβ3 not only recognizes RGD peptide motifs but also binds to other ligands, including ADAM (a disintegrin and metalloprotease) family members, COMP (cartilage oligomeric matrix protein), connective tissue growth factor, ICAM-4 (intercellular cell adhesion molecule-4), and MMP-2 (Seguin et al., 2015). Other integrins that have been identified but less reported include αDβ2, αLβ2, αMβ2, and αXβ2 (Hamidi et al., 2016). Compare with RGD-independent integrins receptors, 8 types of integrins that recognize RGD motifs constitute a most important integrin receptor subfamily instrumental in cancers and their metastasis (Kechagia et al., 2019). However, not all integrins exert a tumor-supporting role in tumorigenesis. Studies have reported that laminin-binding integrins (α3β1 and α6β4) have opposite roles in tumors (Ramovs et al., 2017). Laminin-binding integrins have high affinity to the tetraspanin CD151, which in turn regulate the binding properties of integrin and ECM (Ramovs et al., 2017).

Integrins switch specific ligands from an inactive low avidity state to a high avidity state when binding with them (Shattil et al., 2010). Integrins with altered configuration mediate signal transduction from “outside-in” through physical connection between intracellular domain and actin cytoskeleton, and subsequently activate focal adhesion kinase (FAK) and SRC family kinase (SFK) (Seguin et al., 2015; Cooper and Giancotti, 2019). The activation of intracellular signals can mediate signal transduction from “inside-out,” resulting in increased affinity of integrins and ligands. In conclusion, integrins act as “intermediate contacts” to transmit bidirectional transmembrane signals, thereby affecting the biological functions of cancer cells, including proliferation, metastasis, drug resistance, metabolism and cancer cell stemness (Seguin et al., 2015).

## Integrins and Cancer Metastasis

Cancer metastasis is a complex multi-step process that requires cancer cells to invade from their primary tumor site, survive in the circulation, and eventually colonize on nearby or distant organs (Hoshino et al., 2015). It has gradually become clear that integrins participate in various aspects of these steps in tumor metastasis (Casal and Bartolomé, 2018). Integrins are the main receptors of ECM molecules, and cell adhesion mediated by them is crucial for the spread of cancer cells (Casal and Bartolomé, 2018). In addition, integrins participate in ECM remodeling, provided cancer pre-metastatic niche, and promote survival of circulating cancer cells (CTCs) and colonization of cancer cells in new metastatic sites (Wortzel et al., 2019). However, recent studies have reported that certain integrins, such as integrin α3β1 and α6β4, might exert an inhibitory role in cancer metastasis (Ramovs et al., 2017).

### Integrins Involve in ECM Remodeling

**Figure 1:**
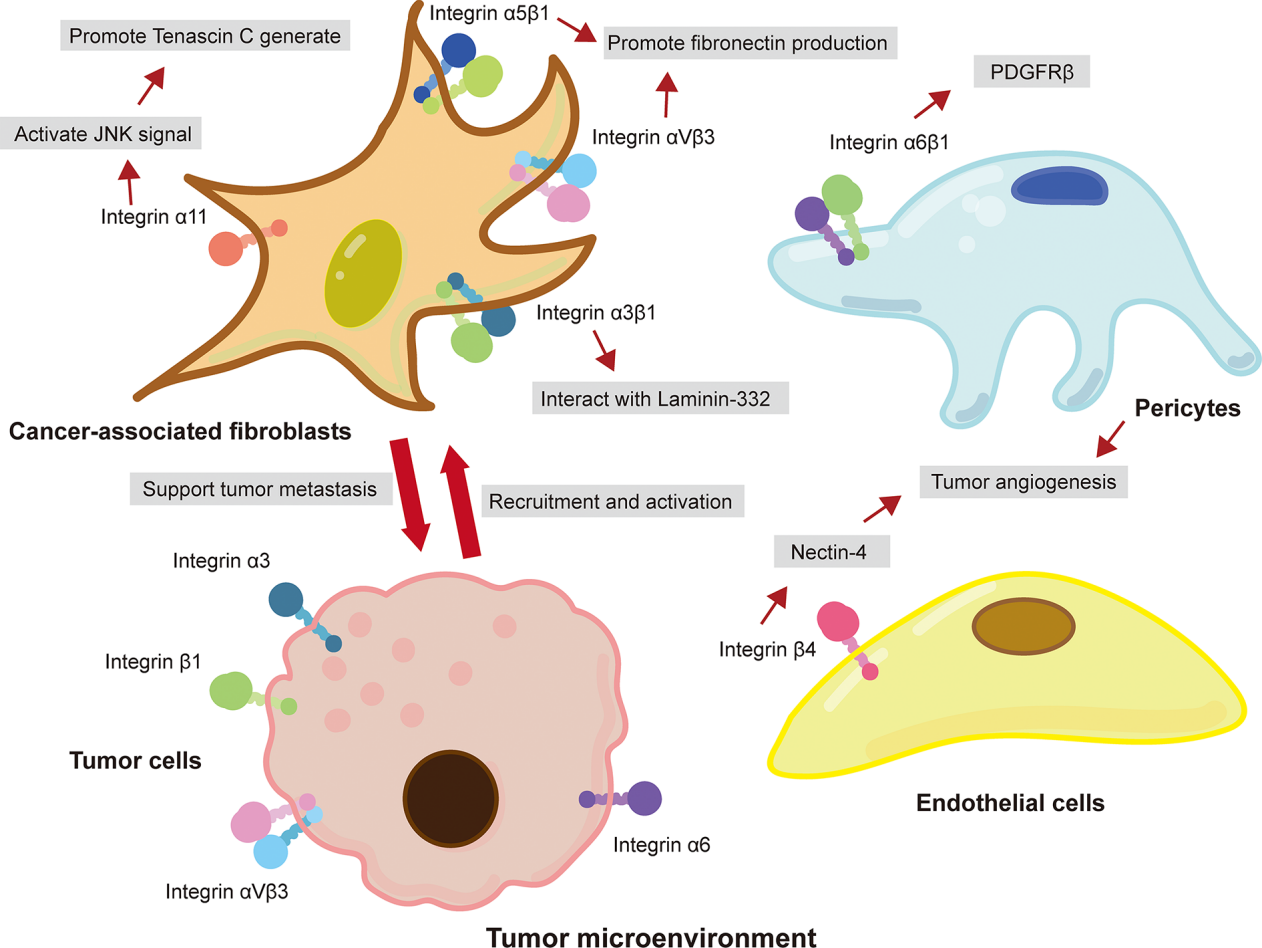
Integrin-mediated crosstalk between cancer cells and tumor-associated stromal cells in the TME promotes cancer metastasis. Integrins expressed on CAFs interacted with ECM and promoted the metastasis of cancer cells. Cancer cells also expressed integrins to recruit and promote the activation of fibroblast. Moreover, pericytes and epithelial cells in TME promoted tumor angiogenesis through the interaction of integrins and ECM.

The TME is rich of ECM components, such as collagens, fibronectin, and laminins, and is the key regulator of cancer metastasis (Hamidi and Ivaska, 2018). In recent years, various studies have reported that integrins are involved in ECM remolding that provide a favorable microenvironment for tumor metastasis (Kai et al., 2019). For example, cancer-associated fibroblasts (CAFs), the most abundant tumor stromal cells in TME, mediated matrix remodeling and matrix deposition through integrins, resulting in increased tumor tissue stiffness (Handorf et al., 2015; Attieh et al., 2017; Jang and Beningo, 2019). CAFs express a variety of integrins, such as integrin αvβ3 (Attieh et al., 2017), α5β1 (Erdogan et al., 2017), and α11 (Primac et al., 2019; Zeltz et al., 2019), that participate in the assembly of fibronectin in ECM and facilitate the conversion of fibronectin matrix to fibronectin and the deposition of CAFs on tumor stroma (Cavaco et al., 2018). Studies have shown that platelet-derived growth factor receptor (PDGFR) is an important intermediate mediator of integrin-mediated ECM remodeling (Erdogan et al., 2017). CAFs aligned fibronectin matrix by increasing non-muscle myosin II and PDGFRα-mediated contractility and traction forces and then converted it to fibronectin by α5β1 integrin (Erdogan et al., 2017). A study performed by Primac and colleagues showed that the crosstalk between CAFs-integrin α11 and PDGFRβ activated downstream JNK signaling pathway, leading to the production of tenascin C (an ECM molecule) (Primac et al., 2019). In addition, pericyte integrin α6β1, a laminin receptor, has been reported to control PDGFRβ and basement membrane structure, which plays a vital role in the stability of tumor blood vessels and the recruitment of pericytes (Reynolds et al., 2017). It is worth noted that tumor cells recruit CAFs and promote their survival by expressing integrins (Peng et al., 2018). Peng et al. (2018) showed that integrin αvβ6 on colon cancer cells induced inactive fibroblasts to become CAFs. Overexpression of integrin α9β1 in breast cancer promoted the recruitment of CAFs (Ota et al., 2014). Briefly, these findings indicated that integrin-mediated ECM remodeling in the TME enables CAFs and cancer cells to communicate with each other, consequently supporting cancer progression and metastasis (**Figure 1**).

### Interaction Between Integrins and ECM Promotes Cancer Invasion and Migration

Cell migration occurs in a variety of physiological and pathological processes, including wound healing, development, induction of immune response, and cancer metastasis (Maritzen et al., 2015). The invasion and migration of tumor cells not only allow cancer cells to spread to distant organs, but more importantly, the increased cell motility permits tumors to grow rapidly by avoiding the steric hindrance and crowding (Waclaw et al., 2015). In the complex regulatory network of tumor metastasis, integrin, as a key regulatory molecule, connects ECM and actin cytoskeleton to support tumor spread (Manninen, 2015). Accumulated studies have shown that integrins interact with a variety of ECM components, activate metastasis-related signaling pathways or molecules, and trigger cancer cell invasion and migration to adjacent tissues. For example, the interaction between integrin α9β1 and tenascin-C promoted the migration of glioblastoma and osteosarcoma cells as well as induced lung metastasis (Sun et al., 2018). Poor cell adhesion mediated by tenascin-C and integrin α9β1 inhibited actin stress fibers, resulting in decreased activity of MKL1 and YAP (Sun et al., 2018). In addition, the combination of integrin αvβ3 and ECM prote*in vitro*nectin upregulates mTOR activity, which overrides the inhibition by hypoxia and facilitates tumor cell invasion (Pola et al., 2013). In oral squamous cell carcinoma, integrin α3 combines with laminin γ2 rich extracellular vesicles (EVs) is absorbed by lymphatic endothelial cells, resulting in enhanced lymphangiogenesis and tumor metastasis to lymph nodes (Wang S. H. et al., 2019). Interestingly, the α5 subunit of integrin α5β1 can be replaced by c-Met to form a c-Met/β1 complex, which has a much greater affinity for fibronectin than α5β1 integrin (Jahangiri et al., 2017). In addition, integrin-linked kinase phosphorylates c-Met, leading to ligand-independent receptor activation (Jahangiri et al., 2017). Crystallography showed that the c-Met/β1 complex could maintain a high-affinity β1 integrin conformation (Jahangiri et al., 2017). The cross-activation of c-Met/β1 integrin complex and its high affinity for fibronectin together drive invasive oncologic processes (Jahangiri et al., 2017).

In addition to activating metastasis-related signaling pathways, the interaction between integrins and ECM has also been reported to promote the intracellular circulation and plasma membrane expression of integrins *via* the endosomal pathway (Novo et al., 2018). The integrins produced through the endosomal pathway can regulate the accumulation and remodeling of proteins in the ECM, thereby facilitating the invasion of tumor cells into adjacent tissues (Novo et al., 2018). Mutant p53 tumor cells showed enhanced invasiveness, characterized by the recycling of Rab-coupling protein (RCP) and diacylglycerol kinase-α (DGKα)–dependent endosomal pathway (Novo et al., 2018). RCP is known for its ability to control integrin recycling (Muller et al., 2009). Mutant p53 tumor cells produced exosomes, which were transmitted horizontally to other tumor cells, and mediated invasiveness and migratory function by activating RCP-dependent integrin recycling (Novo et al., 2018). RCP-driven endocytic recycling of integrin α5β1 promoted actin-related protein 2/3 (ARP2/3) complex-independent ovarian cancer cell migration in 3D ECM rich in fibronectin (Paul et al., 2015). Further research found that ROCK-dependent phosphorylation and FH1/FH2 domain-containing protein 3 (FHOD3)–dependent activation were key mechanisms for cancer cells to mediate invasive migration *via* the RCP-α5β1 integrin pathway (Paul et al., 2015). These findings suggest that integrins play important roles in cancer migration and invasion, mainly through interaction with ECM.

### Integrin Mediates Organ-Specific Metastasis of Cancer Cells

The formation of a pre-metastatic niche is conducive to cancer metastasis to specific sites and colonization of distant organs. Recent evidence has shown that integrins on extracellular vesicles, especially exosomes, promote the establishment of pre-metastatic niche by interacting with cells or ECM at specific tissue sites (Hoshino et al., 2015; Huang et al., 2020). Hoshino and his collages first revealed that exosomal integrins secreted by tumor cell is the decisive factor for tumor organotropic metastasis (Hoshino et al., 2015). Lung-tropic cancer cells secreted α6β1- and α6β4-bearing exosomes preferentially transported to the lungs and were mainly taken up by S100A4^+^ fibroblasts and SPC^+^ epithelial cells (Hoshino et al., 2015). Similarly, liver-tropic cancer cells secreted αvβ5-bearing exosomes, which were preferentially distributed in the liver and were mainly taken up by F4/80^+^ macrophages (Hoshino et al., 2015). Further research found that integrins α6β1 and α6β4 located in the lung bind to laminin in the lung microenvironment, while integrins αvβ5 located in the liver bind to fibronectin (Hoshino et al., 2015). In a study of colorectal cancer, it was found that the primary tumor secreted integrin β1-rich EVs were taken up by resident fibroblasts of remote organs (Ji et al., 2020). Fibroblasts were activated to secrete proinflammatory cytokines (IL-6, IL-8, IL-1β, α-SMA, TGF-β, and CXCL12) to induce the formation of pre-metastatic niche (Ji et al., 2020). It was worth noting that exosomes derived from CAFs also possessed ability to induce the formation of lung pre-metastatic niche (Kong et al., 2019). Exosomal integrin α2β1 of CAFs were uptaken by lung fibroblasts and activated TGF-β signaling pathway, which led to metastasis of salivary adenoid cystic carcinoma (Kong et al., 2019). In a nutshell, tumor exosome integrins are key molecules that mediate tumor cells organ-specific metastasis.

**Figure 2:**
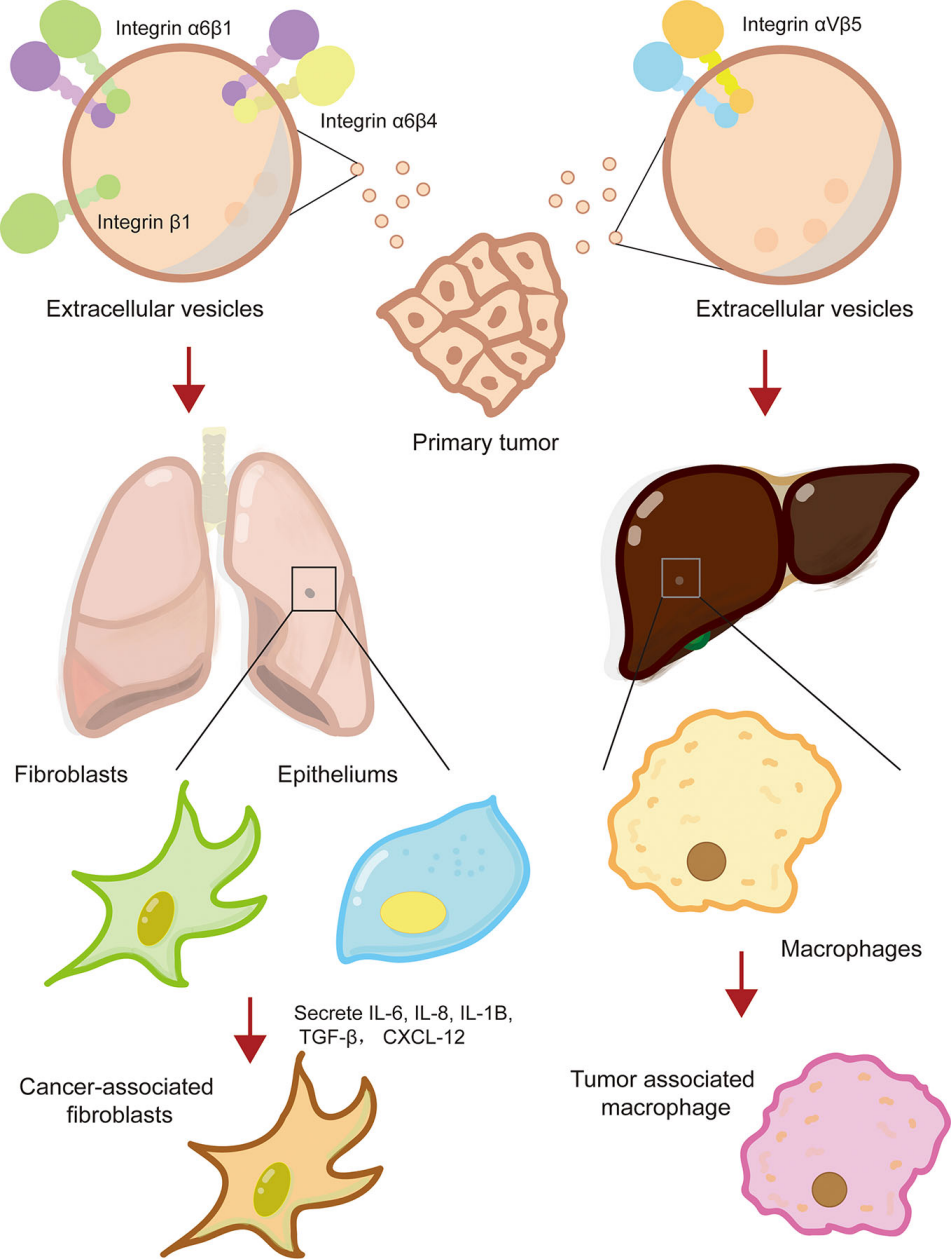
Integrin mediates the formation of cancer pre-metastatic niche. Primary cancer cells secreted extracellular vesicles-containing multiple types of integrins, such that integrin α6β1, α6β4, and αvβ5, which reprogramed lung or live resident fibroblasts, epitheliums, and macrophage to cancer-supporting phenotype and facilitated the formation of cancer pre-metastatic niche.

In addition, it has been reported that the integrins expressed on circulating tumor cells (CTC) also made a significant contribution to organ-specific metastasis of primary tumor cells (Aceto et al., 2014). For example, melanoma is prone to metastasize to different organs in human body, depending on the type of integrins expressed on circulating melanoma tumor cells (Huang and Rofstad, 2018). Melanoma cells expressing integrin β3 tend to metastasize to lungs, while melanoma cells expressing integrin β1 preferentially undergo lymph node metastasis (Vink et al., 1993; Hieken et al., 1999). Additionally, integrin of target organ endothelial cells can help infiltration of CTCs. The underlying mechanism may be related to regulating microvasculature (Huang and Rofstad, 2018). In conclusion, integrins interact with specific ECM components in the tissue microenvironment to promote the formation of pre-metastatic niche, thereby providing a favorable “soil” for cancer cells to metastasis and colonize in specific organs (**Figure 2**).

### The Opposing Roles of Integrins in Cancer Metastasis

Most studies have shown that upregulation/overexpression of integrins is closely associated with cancer metastasis. However, several studies reported that the role of integrins in different types of tumors and different stages of tumor development might be different, meaning that the role of integrins in tumors was complex (Longmate and Dipersio, 2017; Ramovs et al., 2019). For example, in HER2-driven breast cancer, downregulation of integrin α3β1 not only reduced the survival of mice, but also increased tumor growth and vascularization, resulting in an increased burden of lung metastasis (Ramovs et al., 2019). Another study on prostate cancer reported that integrin α3β1 inhibited cancer cell metastasis by regulating Hippo signaling pathway (Varzavand et al., 2016). Integrin α3β1 signals by Abl family kinases to suppress Rho GTPase activity, leading to the inhibition of Hippo pathway, and restrain prostate cancer migration and invasion (Varzavand et al., 2016). Moreover, integrin α9, a molecule related to cell adhesion, mobility and angiogenesis, has been reported to play opposite role in different types of cancers (Zhang et al., 2018; Wang Z. S. et al., 2019). The depletion of integrin α9 in triple-negative breast cancer significantly reduced tumor angiogenesis and metastasis (Wang Z. S. et al., 2019). Mechanistically, knockout of integrin α9 caused integrin-linked kinase (ILK) to relocate from cell membrane to cytoplasm. ILK interacted with protein kinase A (PKA) and inhibited its activity, subsequently increased activity of glycogen synthase kinase 3 (GSK3) and promoted the degradation of β-catenin (Wang Z. S. et al., 2019). However, in HCC, the overexpression of integrin α9 significantly suppressed cancer cell migration *in vitro* and tumor metastasis *in vivo* (Zhang et al., 2018). Thus, attention should be paid to the inhibitory effect of certain integrins in tumors when targeting integrins are used for tumor treatment. More studies are warranted to clarify the mechanisms (**Table 1**).

**Table 1:** Role of integrins in cancer metastasis.

Type of integrins	Cancer cell type/source	Ligand/downstream target	Functions	Ref.
α6β1/α6β4/αvβ5	Breast cancer-Exo/Pancreatic cancer-Exo	S100	Promote the formation of pre-metastasis niche	(Hoshino et al., 2015)
β1	Colorectal cancer-EVs	IL-6, IL-8, IL-1β, α-SMA, TGF-β and CXCL12	Promote the formation of pre-metastasis niche	(Ji et al., 2020)
	Hepatocellular carcinoma-Exo	IL-6/IL-8/NF-κB	Promote the formation of pre-metastasis niche	(Fang et al., 2018)
	Gastric cancer	Galectin-1	Promote migration and invasion	(Kwan et al., 2017)
α2β1	CAFs	TGF-β	Promote the formation of pre-metastasis niche	(Kong et al., 2019)
αvβ3	CAFs	Fibronectin	Promote tumor invasion	(Attieh et al., 2017)
	Breast cancer	Vitronectin/mTOR; IL-8/PI3K/Akt/NF-κB	Promote tumor metastasis	(Pola et al., 2013)
	Pancreatic cancer-EVs	–	Promote tumor metastasis	(Shao et al., 2015)
α5β1	CAFs	Fibronectin	Promote tumor migration	(Erdogan et al., 2017)
	Ovarian cancer	Rab-coupling protein	Promote tumor migration and invasion	(Paul et al., 2015)
α3β1	Pancreatic duct adenocarcinoma	Laminin-332	Promote tumor invasion	(Cavaco et al., 2018)
	Breast cancer	–	Inhibit tumor growth and vascularization	(Ramovs et al., 2019)
	Prostate cancer	Abl/Rho GTPase/Hippo	Inhibit tumor metastasis	(Varzavand et al., 2016)
αvβ6	Colon cancer	TGF-β	Induce fibroblasts to CAFs and promote tumor metastasis	(Peng et al., 2018)
α11	CAFs	PDGFRβ/JNK	Promote tumor metastasis	(Primac et al., 2019)
α6β1	Pericyte	PDGFRβ/Akt-mTOR	Promote tumor angiogenesis	(Reynolds et al., 2017)
α5	Ovarian cancer	–	Promote tumor metastasis	(Gao et al., 2019)
β3	Breast cancer	IL-32/p38-MAPK	Promote EMT and invasion	(Wen et al., 2019)
α9β1	Breast cancer	–	Promote lymphatic metastasis	(Ota et al., 2014)
α3	Lymphatic endothelial cells	Laminin γ2	Promote tumor metastasis to lymph nodes	(Wang S. H. et al., 2019)
β4	Endothelial cells	Src, PI3K, Akt, and iNOS	Promote tumor angiogenesis	(Siddharth et al., 2018)
αx	HUVEC	VEGFR2/VEGF-A/PI3K/Akt/	Promote tumor angiogenesis	(Wang J. S. et al., 2019)
α9β1	Breast cancer	Tenascin-C	Promote migration and metastasis	(Sun et al., 2018)
α9	Breast cancer	ILK/PKA/GSK3/β-catenin	Promote tumor angiogenesis and metastasis	(Wang Z. S. et al., 2019)
	Hepatocellular carcinoma	–	Inhibit tumor migration and metastasis	(Zhang et al., 2018)

Exo, exosomes; CAF, cancer-associated fibroblasts; TGF-β, transforming growth factor-β; α-SMA, α-smooth muscle actin; PDGFRβ, platelet-derived growth factor receptor β; HUVEC, human umbilical vein endothelial cell; VEGF, vascular endothelial growth factor; ILK, integrin-linked kinase; PKA, protein kinase A; GSK3, glycogen synthase kinase 3; “-”, not mention.

## Integrins and Cancer Stemness

Accumulating evidence suggested that crosstalk between integrins and cancer cells activated cancer cell stemness-related signaling pathways, which promoted the transformation of stem-like phenotype and caused the transformation of non-CSCs to CSCs (Seguin et al., 2015). In addition, integrins are biomarkers for normal adult stem and progenitor cells. Recent studies have found that these integrins, such as integrin β1, β4, α6, and α7 also exist on CSCs, that could help identify CSC phenotype (Bierie et al., 2017; Moon et al., 2019; Ge et al., 2020).

## Integrins as Biomarker of Cancer Stem Cells

Cancer cells with overexpression of certain specific integrins exhibit the characteristics of CSCs, suggesting that integrins may become potential biomarkers of CSCs (Haas et al., 2017; Krebsbach and Villa-Diaz, 2017). In fact, integrins β1, α6, and β3 have been found to be overexpressed in normal adult stem and progenitor cells, and recent studies have shown that they are also biomarkers of CSCs. Enrichment of integrin α6 is found in a variety of CSCs, including breast cancer (Brantley et al., 2016), glioblastoma (GSC) (Herrmann et al., 2020), colorectal cancer (Haraguchi et al., 2013) and squamous cell carcinoma (Schober and Fuchs, 2011). Moreover, the overexpression of integrin β4 is associated with enhanced self-renewal ability and chemotherapy resistance in lung cancer cells. Similarly, integrin β4 is overexpressed in GSCs and breast CSCs (Ma et al., 2019). Inhibiting the expression of integrin β4 reduced the self-renewal capacity and tumorigenicity of CSCs (Bierie et al., 2017; Ma et al., 2019). These findings suggest that integrin β4 may be used as a novel biomarker for CSCs. In addition, studies showed that integrin α7 might be a potential biomarker for CSCs (Haas et al., 2017). Integrin α7 is usually up-regulated in CSCs and tumor tissues, which associated with poor clinical characteristics and poor prognosis of patients (Ming et al., 2016; Ge et al., 2020; Lv et al., 2020). Thus, specific integrins can help identify a small subset of the most aggressive and dangerous cancer cells, and provide beneficial information for the diagnosis and prognosis of tumor patients.

### Activation of Integrin Signaling Promotes Cancer Stemness

Recent studies have shown that activation of integrin signaling pathways plays crucial roles in the regulation of cancer cell stemness (Cooper and Giancotti, 2019). Interestingly, current studies indicate that integrins regulate tumor stemness in either a ligand-dependent or a ligand-independent manner. For example, GSCs grown on laminin-coated dishes showed overexpression of integrin αvβ3 and αvβ5, which was related to phosphorylation of FAK and protein kinase B (Paolillo et al., 2018). This result indicates that the interaction of integrins αvβ3 and αvβ5 with laminin is necessary for regulating the stemness of GSCs. Breast CSCs produced Laminin 511, which acted as a ligand for α6Bβ1 integrin and subsequently activated Hippo transducer TAZ to promote the self-renewal ability of cancer cells (Chang et al., 2015). Moreover, colorectal cancer cells cultured on 2D collagen showed enhanced cancer stemness (Wu X. et al., 2019). Mechanistic studies have shown that the interaction of collagen-integrin α2β1 activates the PI3K/Akt/Snail signaling pathway, resulting in enhanced metastasis and stemness of colorectal cancer cells (Wu X. et al., 2019). In pancreatic cancer, integrin αvβ3 interacted with osteopontin on pancreatic stellate cells, which led to the activation of αvβ3-Akt/Erk-FOXM1 (forkhead box protein M1) cascade and promoted CSC-like properties of pancreatic cancer (Cao et al., 2019). However, Seguin et al. (2014) showed that integrin αvβ3 promoted the stemness and drug resistance of lung and pancreatic cancer in a ligand-independent manner. The unliganded integrin αvβ3 had the ability to recruit KRAS and RalB to the plasma membrane of tumor cells, which subsequently led to the activation of TBK1 and NF-κB (Seguin et al., 2014). Indeed, several studies have found that integrins may affect CSCs independent of their capacity to interact with the ECM ligands. Ge et al. (2020) demonstrated that integrin α7 regulated the stemness of HCC by activating PTK2-PI3K-Akt signaling pathway. GSCs used integrin αvβ8 to drive tumor initiation and progression (Guerrero et al., 2017). The activation of integrin αvβ8-TGFβ1 signaling pathway was crucial for the self-renewal of GSCs (Guerrero et al., 2017). Additionally, activation of integrin β1-Notch1 signaling pathway promoted the self-renewal ability and xenograft tumorigenicity of head and neck squamous cell carcinoma (Moon et al., 2019). It is worth noting that integrin α6 and fibroblast growth factor receptor 1 (FGFR1) play a synergistic role in enhancing the expression of glioblastoma stem-related factors and the growth of tumor spheroids (Kowalski-Chauvel et al., 2019). The activation of integrin α6-FAK-STAT3 signaling pathway significantly increased the tumorigenicity and drug resistance of GSCs. (Herrmann et al., 2020). To sum up, integrins activate a variety of downstream signaling pathways in a ligand-dependent or ligand-independent manner, thereby regulating the stemness of tumor cells (**Table 2**).

**Table 2:** Role of integrins in the maintenance of tumor stemness.

Type of integrins	Cancer cell type/source	Ligand/downstream target	Functions	Ref.
α7	Tongue squamous cell carcinoma	FAK	Enhance tumor stemness, EMT	(Ming et al., 2016)
	Hepatocellular carcinoma	PTK2-PI3K-Akt	Enhance tumor stemness	(Ge et al., 2020)
α6	Breast cancer	HIF	Enhance tumor stemness	(Brooks et al., 2016)
	Breast cancer	AhR	Promote mammospheres formation	(Brantley et al., 2016)
	Glioblastoma	FGFR1/FOXM1	Enhance tumor stemness	(Kowalski-Chauvel et al., 2019)
	Glioblastoma	FAK-STAT3	Enhance tumorigenicity and resistance	(Herrmann et al., 2020)
αvβ3	Pancreatic cancer	OPN/Akt-Erk-FOXM1	Enhance tumor stemness	(Cao et al., 2019)
αvβ8	Glioblastoma stem cells	TGFβ1	Promote self-renewal	(Guerrero et al., 2017)
β4	Breast cancer	–	Enhance tumorigenicity	(Ma et al., 2019)
α6Bβ1	Breast cancer stem cells	Laminin 511/Hippo/TAZ	Promote self-renewal	(Chang et al., 2015)

HIF, hypoxia inducible factor; AhR, aryl hydrocarbon receptors; FGFR1, fibroblast growth factor receptor 1; FOXM1, Fokhead Box M1; OPN, osteopontin; “-”, not mention.

## Integrins and Cancer Drug Resistance

More and more studies have elucidated the mechanisms of acquisition and development of cancer drug resistance (Naci et al., 2015; Cruz Da Silva et al., 2019). It is known that resistance to anti-cancer therapies is driven by not only internal factors, such as genetic mutations and epigenetics but also external factors (Seguin et al., 2015). Tumor cells acquired drug resistance by adaptive responses to external stimuli, activation of certain pro-survival signals/anti-apoptotic programs, selection of drug-resistant subpopulations, and alteration of microenvironmental features (Eke and Cordes, 2015; Seguin et al., 2015). Cell adhesion mediated by the interaction between integrins and ECM has been proved to be one of the strategies for tumor cells to evade anti-tumor therapies (Eke and Cordes, 2015).

### Abnormal Activation of Integrin-Driven Signals Leads to Tumor Drug Resistance

Tumor cells often develop resistance to certain targeted drugs (such as tyrosine kinase inhibitors) (Wu and Fu, 2018). One of the reasons is that tumor cells overexpress integrin molecules and activate downstream signaling pathways, thereby triggering cell proliferative signals independent of receptor tyrosine kinase and bypassing the blocking effect of targeted drugs (Kim et al., 2017). It has been reported that activation of integrin β1-driven signal plays a key role in resistance to tumor treatment (Kim et al., 2017; Yang et al., 2018). For example, integrin β1-driven Src-Akt hyperactivation triggered EGFR ligand-independent proliferation signaling in PDAC, resulting in the failure of cetuximab treatment (Kim et al., 2017). Interestingly, Neuropilin-1 (NRP1) physically interacted with active integrin β1, which could be blocked by NRP1 targeting peptide TPP11 (Kim et al., 2017). Therefore, co-targeting EGFR and integrin β1 could produce a synergistic effect, reversing the resistance of PDAC to cetuximab therapy (Kim et al., 2017). In addition, integrin β1 promoted PDAC resistance to gemcitabine by activating the Cdc42 molecule on the PI3Kp110β signaling pathway (Yang et al., 2018). In head and neck cancer, targeting integrin β1 enhanced the sensitivity on cancer cells (Eke et al., 2012; Koppenhagen et al., 2017). c-Abl tyrosine kinase is an important mediator of β1-integrin signaling for radioresistance. AIIB2 (targets integrin β1)/imatinib (targets c-Abl) dual-targeted therapy has radiosensitization effect on tumor cells that grown on 3D laminin-rich ECM cultures and significantly inhibited the DNA damage repair ability of head and neck cancer cells (Koppenhagen et al., 2017). Additionally, a study performed by Eke et al. (2015) showed that simultaneous targeting integral β1 and EGFR had a radiosensitization effect on head and neck cancer. AIIB2 combined with cetuximab and X-ray enhanced cytotoxicity and radiosensitization in various head and neck cancer cells (Eke et al., 2015). Moreover, studies have shown that long-term use of trastuzumab + pertuzumab + buparlisib (PI3K inhibitors) combination treatment in HER2^+^/PIK3CAH1047R transgenic mice with breast cancer produces buparlisib resistant tumors (Hanker et al., 2017; Wang and Xu, 2019). RNA sequencing showed that the genes of ECM and cell adhesion were significantly up-regulated, accompanied by activation of integrin β1/Src signaling pathway (Hanker et al., 2017). It was worth mentioning that this drug-resistant tumor only showed resistance to buparlisib when cells were coated on collagen or re-introduced into mice, while those cells were sensitive to buparlisib *in vitro* 2D culture (Hanker et al., 2017). This result indicated that collagen/integrin β1/Src signal transduction was a key regulatory pathway that mediated the resistance of HER2^+^ breast cancer to anti-HER and anti-PI3K inhibitor combination therapy. In addition to integrin β1, another study found that activation of the integrin α6/Src/Akt signal transduction pathway mediated the resistance of breast cancer cells to tamoxifen (Campbell et al., 2018). Upregulation of integrin α6 was found both in tamoxifen-resistant breast cancer cells and tumor tissue sections from patients who relapsed on tamoxifen treatment (Campbell et al., 2018). In short, integrin is a promising anti-tumor target, and the combination of targeted integrin and other anti-tumor therapies (radiotherapy, chemotherapy, and targeted therapy) has the potential to reverse tumor resistance.

### Crosstalk Between Integrins and ECM Promotes Tumor Drug Resistance

**Figure 3:**
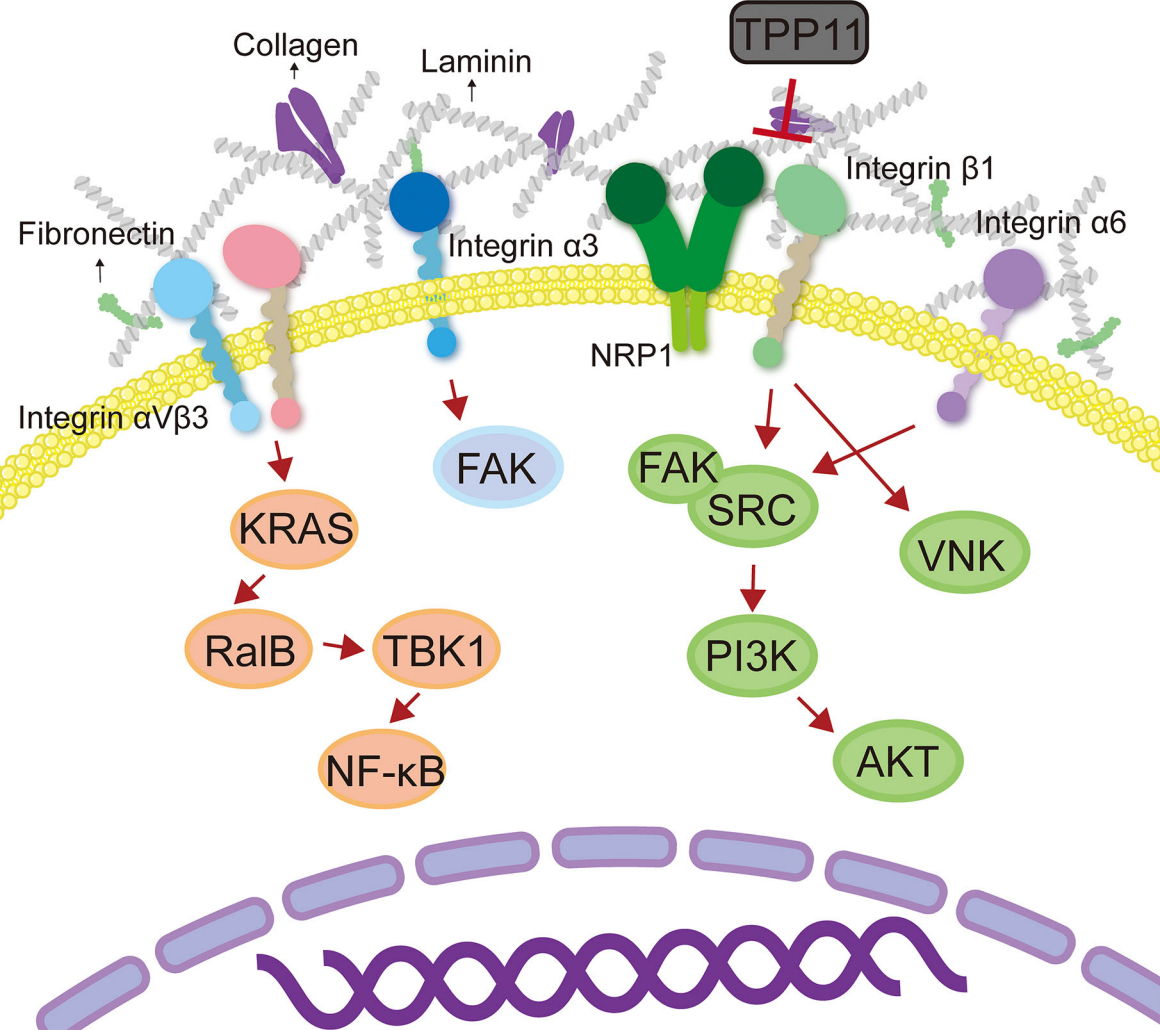
Integrin mediates tumor therapy resistance. Crosstalk between integrins and ECM promoted tumor drug resistance by activating the downstream signaling pathways.

A number of studies have shown that interaction between integrin and ECM is crucial for cancers to develop drug resistance (Azzariti et al., 2016; Jin et al., 2019). Jin et al. (2019) identified that integrin β8 in ECM-based 3D cell culture regulated PDAC resistance to ionizing radiation and cytotoxic drugs. Clinically, patients with HCC often show resistance to sorafenib (Azzariti et al., 2016). Recent studies have shown that HCC resistance to sorafenib is associated with the ECM protein laminin-332 produced by hepatic stellate cells in the HCC TME (Azzariti et al., 2016). The activation of laminin-332-integrin α3 signaling axis reversed the dephosphorylation of sorafenib on FAK, leading to drug resistance (Azzariti et al., 2016). Indeed, ECM stiffening endows tumor cells a strong resistance to chemotherapy. In the collagen-rich microenvironment, the activation of integrin β1 and its downstream effector JNK mediated resistance to sorafenib in triple-negative breast cancer (Nguyen et al., 2014). In addition, resistance to Adriamycin in patients with T-cell acute lymphoblastic leukemia might be due to the interaction between integrin β1 and matrigel that activated the ABCC1 drug transporter (Berrazouane et al., 2019). Glucocorticoid drugs are often used to reduce the toxic and side effects of chemotherapeutic drugs (Chen et al., 2010). However, recent studies have found that dexamethasone increased the levels of integrin β1, α4, and α5 in ovarian cancer cells and enhanced the cancer cells adherent to ECM, thereby mediating resistance to cisplatin and paclitaxel-induced apoptosis (Chen et al., 2010). Another study in ovarian cancer revealed that the combination of ECM protein TGFBI (transforming growth factor beta induced) and integrin β3 mediated the resistance of cancer cells to paclitaxel (Tumbarello et al., 2012). The RGD motif present in the carboxy-terminus of TGFBI is essential for cell adhesion (Tumbarello et al., 2012; Zou et al., 2018) (**Figure 3**). Thus, it would be a promising strategy to reduce or inhibit integrin-mediated ECM stiffness and degradation to achieve homeostasis in ECM, which will increase the penetration of anti-tumor drugs (**Table 3**).

**Table 3:** Role of integrins in cancer therapy resistance.

Type of integrins	Cancer cell type/source	Ligand/downstream target	Functions	Ref.
β1	Pancreatic ductal carcinoma	EGFR/Src-Akt	Promote proliferation; and cetuximab resistance	(Kim et al., 2017)
	Pancreatic ductal carcinoma	Cdc42/PI3Kp110β	Gemcitabine resistance	(Yang et al., 2018)
	Head and neck cancer	c-Abl	Enhance DNA damage repair and radioresistance	(Koppenhagen et al., 2017)
	Head and neck cancer	EGFR	Cetuximab resistance and radioresistance	(Eke et al., 2015)
	Breast cancer	Src/PI3K	Resistance to anti-HER and anti-PI3K inhibitor	(Hanker et al., 2017)
	Breast cancer	JNK	Sorafenib resistance	(Nguyen et al., 2014)
	T-cell acute lymphoblastic leukemia cells	ABCC1	Doxorubicin resistance	(Berrazouane et al., 2019)
α6	Breast cancer	Src-Akt	Tamoxifen resistance	(Campbell et al., 2018)
α3	Hepatocellular carcinoma	Laminin-332/FAK	Sorafenib resistance	(Azzariti et al., 2016)
β3	Ovarian cancer cells	TGFBI	Paclitaxel resistance	(Tumbarello et al., 2012)
	Melanoma stem cell-like cells	–	Doxorubicin and methotrexate resistance	(Zhu et al., 2019)
β8	Glioblastoma-initiating cells	–	Radioresistance	(Jin et al., 2019)
αvβ3	Lung cancer, breast cancer and pancreatic cancer	KRAS/RalB/TBK1/NF-κB	Enhance tumor stemness and resistance	(Seguin et al., 2014)

EGFR, epidermal growth factor receptor; TGFBI, transforming growth factor beta induced; “-”, not mention.

## Current Cancer Therapeutic Strategies by Targeting Integrins: Challenges and Opportunities

The interaction between integrins and their ligands activates downstream signaling molecules and leads to a series of cell biological processes, such as proliferation, differentiation, migration, invasion and development of drug resistance (Huang and Rofstad, 2018). With the elucidation of the mechanisms of integrin-ligand interaction and the encouraging results shown by *in vitro* experiments, integrin targeted drugs and the clinical trials are developed (Stupp et al., 2014). Despite the small number of successful clinical trials, integrins are considered as potential targets for cancer treatment (Hamidi and Ivaska, 2018). More importantly, integrins are also valuable probes in cancer imaging studies and can be used to determine prognosis and therapeutic efficacy (Haas et al., 2017; Huang et al., 2017).

### Main Challenges of Integrins as Therapeutic Targets

Currently, drugs or inhibitors are primarily designed to interfere with integrin-ligand interactions, with the treatment strategy targets integrin itself. However, such treatment strategy has encountered challenges in clinical trials. Multiple clinical studies have shown that integrin-selective inhibitors have not achieved the expected efficacy, whether used alone or in combination with chemoradiation. A multicenter, open-label, phase III study (NCT00689221) evaluated the efficacy of cilengitide (a selective αvβ3 and αvβ5 integrin inhibitor) and standard treatment (temozolomide combined with radiochemotherapy) in newly diagnosed glioblastoma (particularly in tumors with methylated MGMT promoter) (Stupp et al., 2014). Unfortunately, cilengitide has not shown significant benefits for treatment, neither the overall survival nor the prognosis was improved (Stupp et al., 2014; Nabors et al., 2015). Another phase I study (NCT00979862) on cilengitide also yielded frustrating results (Gerstner et al., 2015). Cilengitide plus cediranib was used for the treatment of recurrent glioblastoma showed well tolerance, but the survival and response rate did not warrant further development of this combination (Gerstner et al., 2015). Given the current clinical trial data, cilengitide has been discontinued for the treatment of glioblastoma. However, a phase II clinical trial (CERTO) showed that cilengitide plus cetuximab and platinum-based chemotherapy used in advance NSCLC patients showed potential clinical significance (Vansteenkiste et al., 2015). Compared with the control group, the cilengitide combined group had an improved progression-free survival (PFS) trend (Vansteenkiste et al., 2015). Another phase I study (NCT01118676) for stage III NSCLC patients found that continuous infusion of cilengitide plus chemoradiotherapy showed remarkably favorable clinical response, with a PFS and OS of 14.4 and 29.4 months, respectively (Massabeau et al., 2018). Therefore, although cilengitide has not been further developed as an anti-cancer drug, integrins are still potentially interesting therapeutic targets (Vansteenkiste et al., 2015; Haddad et al., 2017; Yuan et al., 2019).

In addition to cilengitide, several clinical trials have also investigated the efficacy of other integrin-targeted drugs combined with chemotherapeutic drugs, such as abituzumab (a humanized antibody specific for αv integrin) and MINT1526A (an anti-α5β1 monoclonal antibody) (Wirth et al., 2014; Élez et al., 2015; Hussain et al., 2016; Weekes et al., 2018). NCT01008475 was a randomized phase I/II POSEIDON trial that evaluated the efficacy and safety of abituzumab combined with cetuximab plus irinotecan in KRAS wild-type metastatic colorectal cancer (Élez et al., 2015). Although abituzumab did not show improved PFS, it produced an overall survival benefit for patients with high expression of integrin αvβ6 (Élez et al., 2015). In addition, two other clinical trials of castration-resistant prostate cancer (NCT00958477 and NCT01360840) showed that abituzumab was not significantly extended PFS but had potential clinical activity and was worthy of further study (Wirth et al., 2014; Hussain et al., 2016). Moreover, a phase I study (NCT01139723) showed that MINT1526A with or without bevacizumab was well-tolerant and had a preliminary combined effect, although it could not be distinguished from bevacizumab monotherapy (Weekes et al., 2018). In conclusion, the combination of integrin-targeted therapy and chemotherapeutics has potential clinical application value, but there is still a need to develop more effective integrin-specific targeted drugs.

### Potential Treatment Opportunities

Since inhibitors that directly target integrin have not been successfully reflected in clinical treatment, other alternative strategies for inhibiting integrin were developed. Gao et al. (2016) combined integrin-targeted treatment strategy with tumor photodynamic therapy, with the goal of triggering the host immune response to achieve tumor clearance. They used phthalocyanine dye-labeled probes to perform photodynamic therapy on tumors targeted by integrin αvβ6, which significantly inhibit lung metastasis in the mouse breast cancer model (Gao et al., 2016). In addition, the treatment promoted the maturation of dendritic cells and the killing activity of CD8^+^ T cells (Gao et al., 2016). Combining integrin-targeted therapy with cancer immunotherapy is another potential strategy. Kwan et al. (2017) prepared an integrin-binding peptide fused to the antibody Fc-domain and used it together with the engineered mouse serum albumin/IL-2 fusion, which significantly improve the survival of various types of tumor mouse models. This treatment strategy promoted the activation of CD8^+^ T cells and natural killer cells by activating the host immune system, rather than blocking the integrin function to achieve therapeutic effects (Kwan et al., 2017).

The overexpression of integrin in cancer cells makes it a promising molecular target in integrin targeting-probes for non-invasive medical imaging and development of biomarkers (Cooper and Giancotti, 2019; Xiao et al., 2019). Recently, the development of radiotracers for integrin targets was used to predict the overall survival and prognosis of patients (Huang et al., 2017). An early phase I clinical trial (NCT04289532) was the first to use 99mTc-RWY, a radiotracer targeting integrin α6, to conduct SPECT (single-photon emission computed tomography) imaging in breast cancer patients. Moreover, Huang et al. (2017) used the integrin α2β1 targeting 68Ga-DOTA-A2B1-PET (positron emission tomography) imaging to identify the phenotypes of aggressive lung cancer and monitor drug responses. Interestingly, PET imaging of the RGD motif-containing αvβ6 integrin-binding peptides SFLAP3 also showed the potential for diagnosing head and neck squamous cell carcinoma (Roesch et al., 2018). Other similar radiotracers include RDG-K5 PET/CT for integrin αvβ3, which has the potential to identify patients with incomplete response to concurrent chemoradiotherapy (Chen et al., 2016). In addition to being a molecular targeted probe, integrins can also be used for cancer diagnosis and prognosis by directly detecting the expression level of specific integrins in serum or tissues. For example, integrin αvβ3 has been shown to be a potential diagnostic and prognostic biomarker in a variety of cancers, including gastric cancer (Böger et al., 2015), breast cancer (Radwan et al., 2019), glioblastoma (Zhang et al., 2019), and lung cancer (Schniering et al., 2019). It is worth noting that new anti-cancer therapies targeting integrins using nanoparticles as carriers are emerging. The treatment strategy is to use integrin-specific ligands to engineer nanoparticles, thereby increasing their affinity for cancer cells (Wu P. H. et al., 2019). In summary, integrins have shown great potential in the diagnosis, prognosis, and treatment of cancer. However, more clinical trials are needed for further verification.

## Conclusions

As a cell membrane receptor, crosstalk between integrin and ECM is crucial for cancer metastasis, maintenance of cancer stemness, and drug resistance. Integrin-targeted treatment strategy is an emerging cancer treatment concept. Because of the remarkable therapeutic effect of targeting integrin in preclinical research, more *in vitro* and preclinical studies are warranted to fully understand the mechanisms of integrin-mediated biological behavior of cancer cells, which will facilitate further development of drugs targeting integrin signaling pathways.

## Author Contributions

CS, JL, and LZ wrote the first draft of the manuscript. HW, FW, YT, YW, QG, and JL organized the structure of the manuscript. JZ, YY and YL contributed conception of the work. All authors contributed to the article and approved the submitted version.

## Funding

This work was supported by National Natural Science Foundation of China (U1903126, 81773888, and 81902152), Natural Science Foundation of Guangdong Province (2020A151501005 and 2020A1515010605), Fund from Guangzhou Institute of Pediatrics/Guangzhou Women and Children’s Medical Center (nos. YIP-2018-031 and IP-2018-012).

## Conflict of Interest

The authors declare that the research was conducted in the absence of any commercial or financial relationships that could be construed as a potential conflict of interest.
